# Supplementary material for: Instantaneous movement-unrelated midbrain activity modifies ongoing eye movements
Source: eLife. 2021 May 6;10:e64150. doi: 10.7554/eLife.64150 (PMC8143798; doi:10.7554/eLife.64150)
Supplement: Transparent reporting form [file elife-64150-transrepform.docx]

***eLife’s* transparent reporting form**

We encourage authors to provide detailed information *within their submission* to facilitate the interpretation and replication of experiments. Authors can upload supporting documentation to indicate the use of appropriate reporting guidelines for health-related research (see [EQUATOR Network](http://www.equator-network.org/%20)), life science research (see the [BioSharing Information Resource](https://biosharing.org/)), or the [ARRIVE guidelines](http://www.plosbiology.org/article/info:doi/10.1371/journal.pbio.1000412) for reporting work involving animal research. Where applicable, authors should refer to any relevant reporting standards documents in this form.

If you have any questions, please consult our Journal Policies and/or contact us: [editorial@elifesciences.org](mailto:editorial@elifesciences.org).

**Sample-size estimation**

- You should state whether an appropriate sample size was computed when the study was being designed
- You should state the statistical method of sample size computation and any required assumptions
- If no explicit power analysis was used, you should describe how you decided what sample (replicate) size (number) to use

Please outline where this information can be found within the submission (e.g., sections or figure legends), or explain why this information doesn’t apply to your submission:

We did not explicitly estimate sample size. However, repeating the experiments on 2 different monkeys, with consistent results, is in line with the standard of 2 monkeys per study (due to compromises associated with the 3R principle for the use of non-human primates in research). Some of our analyses (e.g. Figure 5) used as statistical observation the single eye movement (with some number of intra-movement spikes), so all the data across monkeys and neurons were combined to describe the effects. Our figures are based on hundreds of eye movements per shown condition, and we consistently reported either 95% confidence intervals or one standard error of the mean to highlight differences between conditions. Whenever we split the data into different subgroups, for the most extreme conditions, we ensured having at least 15 measurements (e.g. Figure 4A). All these technical precautions increase our confidence in our results.

**Replicates**

- You should report how often each experiment was performed
- You should include a definition of biological versus technical replication
- The data obtained should be provided and sufficient information should be provided to indicate the number of independent biological and/or technical replicates
- If you encountered any outliers, you should describe how these were handled
- Criteria for exclusion/inclusion of data should be clearly stated
- High-throughput sequence data should be uploaded before submission, with a private link for reviewers provided (these are available from both GEO and ArrayExpress)

Please outline where this information can be found within the submission (e.g., sections or figure legends), or explain why this information doesn’t apply to your submission:

The analyses on the neuronal data of Experiment 1 and 2 were performed on an already published dataset (Chen et al. 2015; Khademi et al., 2020). We analyzed 84 sessions (one per neuron with the only exception of one neuron for which two different stimulus locations were tested in the receptive field) in Experiment 1 and 55 (one per neuron) in Experiment 2 across two monkeys. For Experiment 3, we collected an additional 53 sessions. Out of the 53 sessions, SC electrode penetrations were performed either the rostral or caudal SC (23 and 17 sessions, respectively) or simultaneously (13 sessions), one in the rostral SC and one in the caudal SC. The large number of neurons recorded gives us confidence on the reliability of the data. The “Data analyses” section of Methods describes how each analysis was performed indicating the precise time windows of analysis and which statistics were used. Eye movement flagging and spike sorting were both performed in a semi-automatic fashion, with always the supervision of an experienced researcher. After careful preprocessing of the eye movement and neural signals, all the data entered in the analysis.

**Statistical reporting**

- Statistical analysis methods should be described and justified
- Raw data should be presented in figures whenever informative to do so (typically when N per group is less than 10)
- For each experiment, you should identify the statistical tests used, exact values of N, definitions of center, methods of multiple test correction, and dispersion and precision measures (e.g., mean, median, SD, SEM, confidence intervals; and, for the major substantive results, a measure of effect size (e.g., Pearson's r, Cohen's d)
- Report exact p-values wherever possible alongside the summary statistics and 95% confidence intervals. These should be reported for all key questions and not only when the p-value is less than 0.05.

Please outline where this information can be found within the submission (e.g., sections or figure legends), or explain why this information doesn’t apply to your submission:

We show raw data both in Figure 2 (examples of single microsaccade timing after stimulus onset, Experiment 1; example of eye movement trajectories and velocities for one monkey, Experiment 1), Figure 5 and Figure 5 – supplement figure 1 (raster plot with all the spikes collected within a critical interval across monkeys and neurons, Experiment 1), and Figure 7 and Figure 7 - supplement figure 1 (raster plots with all the spikes collected within a critical time window in two neurons). Four other example neuron receptive fields and firing rates are presented in Figure 9 and Figure 10. Statistical analysis techniques are described in a dedicated “Statistics” section in Methods. We provided the exact p-values in the Results text. Measures of central tendency and dispersion are all defined in the figure legends. Confidence intervals are also shown in the figures, and defined in the figures and legends.

(For large datasets, or papers with a very large number of statistical tests, you may upload a single table file with tests, Ns, etc., with reference to sections in the manuscript.)

**Group allocation**

- Indicate how samples were allocated into experimental groups (in the case of clinical studies, please specify allocation to treatment method); if randomization was used, please also state if restricted randomization was applied
- Indicate if masking was used during group allocation, data collection and/or data analysis

Please outline where this information can be found within the submission (e.g., sections or figure legends), or explain why this information doesn’t apply to your submission:

No group allocation was needed in this study.

**Additional data files (“source data”)**

- We encourage you to upload relevant additional data files, such as numerical data that are represented as a graph in a figure, or as a summary table
- Where provided, these should be in the most useful format, and they can be uploaded as “Source data” files linked to a main figure or table
- Include model definition files including the full list of parameters used
- Include code used for data analysis (e.g., R, MatLab)
- Avoid stating that data files are “available upon request”

Please indicate the figures or tables for which source data files have been provided:

We can upload the data associated with figures upon acceptance.
